# Insights into the Landscape of Alphavirus Receptor and Antibody Interactions

**DOI:** 10.3390/v17071019

**Published:** 2025-07-21

**Authors:** Shishir Poudyal, Abhishek Bandyopadhyay, Richard J. Kuhn

**Affiliations:** 1 Department of Biological Sciences, Purdue University, West Lafayette, IN 47907, USA; spoudyal@purdue.edu (S.P.); bandyoa@purdue.edu (A.B.); 2 Purdue Institute of Inflammation, Immunology, and Infectious Disease, Purdue University, West Lafayette, IN 47907, USA

**Keywords:** alphavirus, attachment factors, receptors, antibodies

## Abstract

Alphaviruses engage a diverse array of attachment factors and receptors during viral entry, resulting in a broad host range and disease spectrum, and thus presenting them as a major global public health concern. The development of effective antivirals against these arboviruses relies on a comprehensive understanding of the molecular interplay between these viruses and host cell factors, as well as the wide range of immune responses that ensue following viral infection. In this review, we present the current understanding of the complex landscape of alphavirus interaction with attachment factors and entry receptors, some of which are characterized structurally, while others are characterized biochemically. Additionally, we provide an overview of the molecular bases of epitope recognition by neutralizing and non-neutralizing antibodies against alphaviruses, and how icosahedral symmetry influences these interactions, such as occupancy and neutralization potency. We further discuss the structural bases of epitope recognition of a few pan-alphavirus antibodies, their potential therapeutic implications, and offer future perspectives on the development of effective therapeutics against clinically relevant alphaviruses.

## 1. Introduction

Alphaviruses are arboviruses that cause a wide range of diseases in humans and other animals. Historically, alphaviruses have been classified into two major groups, termed Old World alphaviruses and New World alphaviruses, based on their geographical origins. Examples of some Old World alphaviruses are Chikungunya virus (CHIKV), Sindbis virus (SINV), Ross River virus (RRV), and Mayaro virus (MAYV), while Eastern equine encephalitis virus (EEEV), Western equine encephalitis virus (WEEV), and Venezuelan equine encephalitis virus (VEEV) constitute New World alphaviruses [1,2]. The Old World and the New World alphaviruses also cause different pathologies in infected individuals, with the former causing mainly fever, rash, and debilitating arthritis and, therefore, are termed arthritogenic; the latter group causes severe encephalitis and is termed encephalitic. In infants and elderly patients, encephalitic alphaviruses can cause severe encephalitis with high mortality rates, and the survivors often suffer lifelong neurological sequelae. Additionally, the encephalitic alphaviruses can be aerosolized and, therefore, pose a significant bioweapon threat [3]. The FDA has recently approved two vaccines against CHIKV for human use (https://www.cdc.gov/chikungunya/prevention/chikungunya-vaccine.html and https://www.fda.gov/vaccines-blood-biologics/vimkunya (accessed on 30 May 2025)). However, there are no approved therapeutics or vaccines against the encephalitic alphaviruses.

## 2. Alphavirus Structure and Influence on Receptors and Antibodies Binding

**Figure 1:**
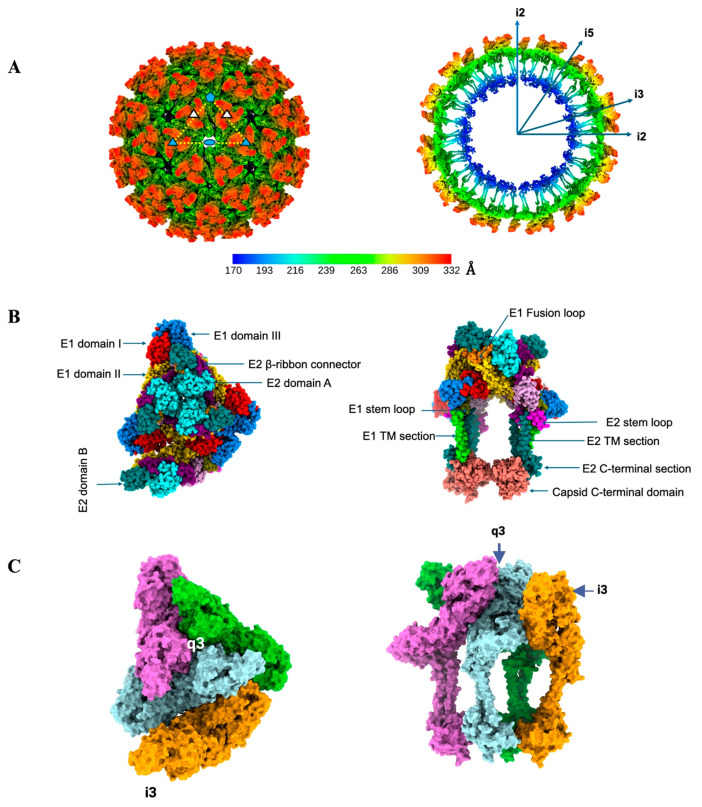
Structural organization of an alphavirus. (**A**) *Left*, Icosahedral reconstruction of EEEV (20). The asymmetric unit is indicated with a white dotted triangle and the icosahedral five-, three- and two-fold symmetry axes are shown with blue pentagon, triangle and oval, respectively. The q3 spikes are indicated with white triangles. *Right*, central slice of the volume on the left. The symmetry axes are indicated. The color key represents the radial coloring of the volume in Å. The N-linked glycans at E1 Asn-134 and E2 Asn-315 are shown as sticks. (**B**) *Left*, Atomic model of the asymmetric unit viewed from the top. The asymmetric unit consists of one q3 spike and one arm of the i3 spike. The different domains of E1 and E2 and the Capsid protein are colored according to the following scheme. E1 domain I red, domain II gold, fusion loop dark orange, domain III blue, stem loop olive, TM (transmembrane region) lime green. E2 domain A cyan, domain B teal, β-ribbon connector purple, domain C plum, stem loop magenta, and TM dark cyan. The capsid is in salmon. *Right*, side view of the model shown in left. The different domains are indicated. The glycans on E1 and E2 are shown as sticks. (**C**) *Left and Right*, surface representation of the model shown in (**B**) in the same top view and slightly rotated side views, respectively. The q3 spikes are colored lime green, powder blue, and orchid, whereas i3 spike is colored orange.

**Figure 2:**
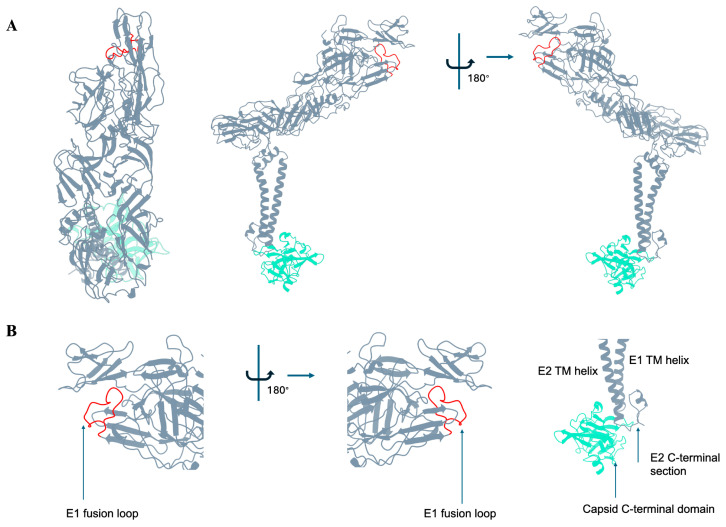
Structural organization of E1/E2 heterodimer. (**A**) *Left*, Top view of the E1/E2 heterodimer in cartoon representation. The E1/E2 heterodimer is colored in slate gray, the E1 fusion loop is colored red, and the Capsid is colored medium aquamarine. Middle and *right*, side views of the E1/E2 heterodimer. (**B**) *Left and middle*, zoomed-in view of the interaction of the E1 fusion loop with E2 domain B. The fusion loop is indicated. The E2 domain B clamps onto the underlying E1 fusion loop. *Right*, E2 C-terminal tail interacts with a hydrophobic cavity in the C-terminal domain of the Capsid protein, which imparts additional stability to the particle. The transmembrane (TM) helices of E1 and E2 are indicated.

The alphavirus virion consists of a positive-sense single-stranded RNA as its genomic material, enclosed by 240 copies of capsid protein arranged as an icosahedral shell with T = 4 quasi-symmetry [4]. The nucleocapsid is further encapsulated by a glycoprotein layer formed by two single-pass transmembrane proteins, E1 and E2, embedded in a host-derived lipid bilayer. The subsequent trimerization of E1 and E2 heterodimers results in 80 spikes arranged as an icosahedron with T = 4 quasi-symmetry (Figure 1). The approximately 35 kDa capsid protein is composed of N- and C-terminal domains. The N-terminal domain binds the single-stranded RNA and is predicted to be an intrinsically disordered domain, whereas the C-terminal domain adopts a chymotrypsin-like fold [4,5]. The C-terminal tail of each E2 interacts with a hydrophobic cavity in the C-terminal domain of the capsid, stabilizing this double-layered icosahedral structure (Figure 2).

The E1 protein lies roughly parallel to the viral membrane, and the E2 protein lies on top of E1 and forms the outermost layer of the virus. Much of E1 is also occluded from the surface due to the way E2 positions itself on E1. E1 is the fusion protein, and E2 is mostly responsible for cellular attachment and receptor engagement [4,6]. Additionally, recent advances in the structures of several alphaviruses in complex with cell surface receptors show that some residues on E1 also interact with receptors [7,8]. E1 and E2 each consist of three domains, of which E1 domains I, II, and III belong to the β-barrel type domain, whereas E2 domains A, B, and C belong to immunoglobulin (Ig)-like domain. E2 ectodomain consists of domains A, B, and C, with domain B being an insertion into domain A. These three domains are connected with flexible linkers termed beta-ribbon connectors. Similarly, the E1 ectodomain consists of domains I, II, and III, with domain II being an insertion into domain I. Domain II harbors the fusion loop, which is covered by E2 domain B (Figure 2). Additionally, there are two other transmembrane proteins, 6K and TF (Transframe), that are incorporated in virions in non-stoichiometric amounts and have not yet been structurally characterized [9,10].

Alphavirus infection initiates with the binding of viral glycoproteins to specific host cell receptors or attachment factors which concentrate the virus on the cell surface, followed by receptor engagement triggering clathrin-mediated endocytosis. Acidification of the endosome facilitates fusion between the viral envelope and the endosomal membrane, resulting in the release of the viral RNA genome into the host cell cytoplasm [11]. The icosahedral structure provides structural rigidity to the virus and an energy-efficient way of packaging the genome. Further, the structural arrangement provides a highly organized and repetitive arrangement of envelope proteins, which dictates the spatial distribution of receptor-binding sites on the viral surface, crucial for virus–host interaction [12]. Moreover, the icosahedral arrangement of the E1/E2 glycoproteins provides unique surface topography, such as spikes, clefts, and comparatively flat and curved surfaces, that control its ability to bind receptors and antibodies [13]. The presence of quasi-equivalent sites on alphaviruses, owing to T = 4 symmetry, results in slightly different structural and chemical environments. Even though the symmetrical arrangement of the viral envelope proteins provides an enticing platform for ligand binding, steric constraints arising out of the quasi-equivalence of the icosahedral arrangement of the glycoprotein shell in alphaviruses often govern the ligand occupancy of available sites. For bivalent or multivalent binders (antibodies or oligomeric forms or receptors), avidity effects and the ability to form intra-virion or inter-virion crosslinking often impact biological outcomes. During the initial steps of viral entry, high-affinity virus-receptor complexes typically lead to efficient endocytosis. However, in some cases, receptors with multiple domains capable of individually interacting with the viral surface with low affinity often utilize simultaneous binding of those domains to the multivalent viral surface, leading to avidity-driven high-affinity complex formation, thereby enhancing the internalization of the virus [13,14]. The requirement of these kinds of interactions to cross the threshold could also lead to higher specificity of infection, i.e., to sites where relevant receptors are expressed, in other words, viral tropism. These could be easily explained in terms of virus-engaging attachment factors like heparan sulfate (HS). HS by itself cannot trigger the entry into the host cells, but alphaviruses like EEEV, SINV, and RRV are concentrated by HS on relevant cells, which in turn engage true receptors for entry, which could be explained in terms of the proximity effect [7].

Viral entry is a complex and highly coordinated multistep phenomenon that involves the engagement of several host factors by viral glycoproteins for a productive infection. The involvement of attachment factors, which help concentrate the virus, and receptor engagement ultimately leads to viral entry into the host cell. Further detailed functional and structural studies are required to elucidate this further.

## 3. Cellular Receptors and Attachment Factors

**Figure 3:**
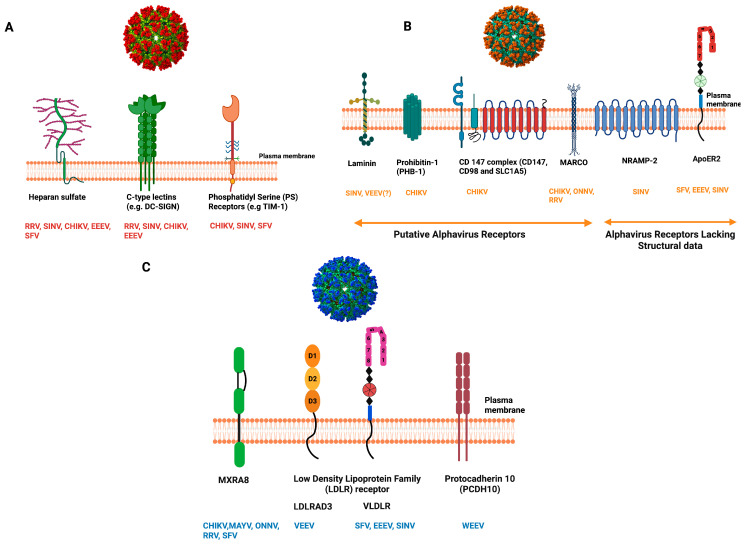
Alphavirus interactions at the host cell surface. Alphaviruses concentrate at the host cell surface utilizing several host cell surface moieties called attachment factors like HS, DCSIGN, and PS. The attachment facilitates them for the next stage of entry, i.e., binding to entry receptors, which triggers their internalization. (**A**) *Top left*, The attachment factors engaged in alphavirus binding are listed along with the virions they engage in. (**B**) *Top right*, All putative and confirmed receptors for which direct binding/structural data are absent are grouped. (**C**) *Bottom Right*, All the receptors for which structural information about the virus-receptor complex is known are grouped. Viruses color coded to indicate attachment factors/receptor utilization. Figures not drawn to scale. RRV—Ross River, SINV—Sindbis, CHIKV—Chikungunya, SFV—Semliki Forest, MAYV—Mayaro, ONNV—O’nyong-nyong, EEEV—Eastern, VEEV—Venezuelan, WEEV—Western Equine Encephalitis Virus. HS—Heparan sulfate, DC-SIGN—Dendritic cell-specific intercellular adhesion molecule-3-grabbing, TIM-1—T cell immunoglobulin mucin domain 1, NRAMP2—Natural resistance-associated macrophage protein 2 nonintegrin, Mxra8—matrix remodeling associated protein 8, LDLRAD3—Low-density lipoprotein receptor class A domain-containing protein 3, VLDLR—Very low-density lipoprotein, ApoEr2—Apolipoprotein E receptor-2.

Alphavirus entry into host cells begins with initial attachment to target cells, followed by endocytosis of the receptor-bound virion. The attachment factors, which alone cannot trigger viral entry, concentrate infectious particles on the cell surface, allowing the authentic receptors to engage them [6,15]. Productive viral entry requires a synergistic involvement of attachment factors and receptors; their absence can diminish viral infectivity [16]. Over the past several decades, extensive research has identified many of the attachment factors and receptors involved in alphavirus entry [17,18,19,20]. Various biochemical, genetic, structural, and functional approaches have been employed to identify and establish the complex interplay between viral glycoproteins and host cell surface receptors and attachment factors, as summarized in Figure 3.

### 3.1. Alphavirus Attachment Factors

Attachment factors provide an initial docking site for alphaviruses, readying their entry into host cells. The initial interactions, albeit of lower affinity, bring the viruses proximal to the bona fide receptors, which will then trigger entry. The attachment factors indicated in alphavirus entry have been broadly categorized into: heparan sulfate, C-type lectin, and phosphatidyl serine receptors [16]. In this review, we classify and discuss these factors based on the availability of structural data regarding their interaction with alphaviruses.

#### 3.1.1. Attachment Factors Without Structural Characterization with Alphaviruses

In this section, we present attachment factors demonstrated to aid in alphavirus entry; however, structural characterization in complexes with these viruses is lacking.

C-type lectins (CTL):

C-type lectins constitute a large family of calcium (Ca^2+^)-dependent glycan-binding proteins characterized by one or more C-type lectin folds, which are structurally conserved and commonly referred to as carbohydrate-recognition domains (CRDs) [21]. Alphavirus glycoproteins, particularly those of mosquito origin, frequently exhibit a higher abundance of high-mannose N-linked oligosaccharides compared to mammalian cells. C-type lectins, such as DC-SIGN and L-SIGN, preferentially bind these high-mannose structures. Consequently, the CRDs of C-type lectins on host cell surfaces can directly interact with the mannose-rich glycans on the viral envelope protein, facilitating viral attachment [21]. SINV productively infected otherwise refractory cells expressing DC-SIGN or L-SIGN as attachment receptors, suggesting these lectins may contribute to the initial cell and tissue tropism of alphaviruses by enhancing viral binding and, subsequently, entry [19]. It was speculated that the interaction involved carbohydrate-mediated binding between the viral E2/E1 glycoproteins and the lectin CRDs, although no structural data is available. However, cryo-EM analysis of an unrelated flavivirus, dengue virus (DENV) complexed with DC-SIGN-CRD, revealed that a single DC-SIGN CRD monomer binds two Asn67 glycosylation sites on adjacent DENV E glycoproteins within each icosahedral asymmetric unit, leaving one Asn67 site unoccupied [22]. Drawing parallels with the DENV-DC-SIGN structure, it is plausible that N-glycosylated residues on the E2 glycoprotein are involved in alphavirus binding. However, given the arrangement of heterodimers, the stoichiometry and binding residues may vary.

PS receptors

Phosphatidylserine (PtdSer) is an anionic phospholipid molecule predominantly located on the inner leaflet of the plasma membrane. Enveloped viruses that bud through the plasma membrane incorporate these negatively charged phospholipids in their lipid bilayer, which they later use for ligand binding, replication, and modulating the immune response [23]. Viruses expose PtdSer to mimic apoptotic bodies, a process called apoptotic mimicry, tricking cells into engulfing them. PtdSer receptors (PVEERs) enhance entry for various enveloped viruses, providing a broad, low-cost entry mechanism and potentially facilitating glycoprotein/receptor interactions by promoting initial attachment.

Several PS receptors can recognize and bind to PtdSer exposed on the viral lipid bilayer and aid in viral attachment: (a) TIM receptors: T-cell immunoglobulin and mucin domain-containing proteins (e.g., TIM-1, TIM-4). The role of TIM-1 as an attachment factor for CHIKV, SINV, RRV, and EEEV has been demonstrated utilizing E1/E2 pseudotyped viruses [18,22]; and (b) TAM receptors: Tyro3, Axl, and MerTK receptors of the TAM family, SINV has been shown to engage the Axl receptor to bind PtdSer mediated by Gas6. Gas6 essentially bridges the Axl receptor to the viral envelope PtdSer [24].

#### 3.1.2. Attachment Factors Structurally Characterized with Alphaviruses

This section discusses the only attachment factor that has been characterized structurally and have been implicated in alphavirus entry.

Heparan sulfate (HS):

Many alphaviruses utilize heparan sulfate (HS), a highly sulfated glycosaminoglycan (GAG) found on cell surfaces and within the extracellular matrix, as an initial attachment factor. Their ability to bind HS can arise naturally or through cell culture adaptation (14). Arthritogenic alphaviruses, such as CHIKV, RRV, and SINV, have been observed to bind HS following cell culture adaptation. This enhanced binding is attributed to specific amino acid substitutions within their envelope proteins. For instance, in CHIKV, the E2-A domain residue G82R substitution resulted in increased HS binding, albeit with a concomitant reduction in in vivo pathogenicity [25]. Similarly, the N218K substitution in the E2-B domain of RRV led to high-titer viruses [26] and enhanced HS-dependent infectivity in the N218R variant [25]. Non-passaged EEEV isolates naturally employ HS as an attachment factor. This ability is directly correlated with neurovirulence, as evidenced by substitutions that abolish HS binding, resulting in diminished neurovirulence [18]. Conversely, EEEV isolates exhibiting increased HS binding demonstrate an opposite effect on pathogenicity when inoculated subcutaneously vs. intracranially [27]. Interestingly, alphaviruses, like SINV and VEEV, having cell culture-adapted positively charged substitutions in the HS-binding region that increase heparin binding, exhibit rapid clearance from the blood and reduced virulence in mice [27,28,29]. The increased HS binding in some of these viruses causes their restriction at the inoculation site, as well as rapid clearance by the liver, leading to shorter half-lives and preventing successful infection of the central nervous system. However, the ability to bind heparin is not the only factor that determines in vivo virulence [30].

An earlier cryo-EM structure of the RRV N218R mutant in complex with heparin revealed the binding of four heparin molecules on the distal tip of each E2 protein in an asymmetric unit [31]. Mutagenesis analysis of conserved lysine residues in E2-domain A [18] and an earlier cryo-EM analysis of native EEEV from our group revealed three residues (K71, K74, and K77) that form the binding interface with the highly negatively charged HS molecule [6]. However, a more recent high-resolution cryo-EM structure of EEEV complexed with heparan polysaccharide (Hp) from our group demonstrated that HS has four contact points per trimeric spike, with three of them along the q3 axis and one along the vertex of the spike involving several basic amino acids as the interacting interface [32]. The peripheral HS binding sites were adjacent to the E2 β-connector and surrounded by basic residues: His5 and 155, Arg156, and Lys157 of E2, whereas the axial binding site was proximal to His82, Arg84, His114, and Arg119. It was also noted that these residues were not conserved among different alphaviruses, thus indicating different binding interfaces among different alphaviruses [32]. A recent study on Getah virus (GETV) using mouse models demonstrated the requirement of E2 R253-mediated HS binding that sits closer to Lys252 and His256 for virulence, pointing to a new HS-binding motif [33].

Heparan sulfate binding presents a complex but dynamic aspect of alphavirus biology that needs further scrutiny. The differences in HS-engaging residues determined from mutational vs. cryo-EM analyses present another aspect that needs to be addressed.

### 3.2. Alphavirus Receptors

To qualify as a bona fide viral receptor, a cell surface protein must satisfy the established gold standard criteria: (a) direct interaction with a viral protein, (b) induction of virus internalization, (c) inhibition of viral infection in the presence of receptor blocking agents such as antibodies or decoy receptors, and (d) correlation of viral tropism with receptor expression levels [6,15]. Several alphavirus receptors have been characterized using various genetic, biochemical, and functional assays. The advent of CRISPR/Cas-9 and its utilization for understanding alphavirus entry has led to the discovery of newer receptors. These studies, along with high-resolution cryo-EM structures of alphavirus-receptor complexes, will aid in the development of newer therapeutics and vaccines.

#### 3.2.1. Receptors Without Structural Characterization as Complexes with Alphaviruses

In this section, we present different proteins identified as receptors for different alphaviruses but await further in vivo characterization and/or structural characterization as complexes with viruses. Those receptors that have not met the gold standard criteria are discussed under putative receptors.

- Putative Receptors

Laminin receptor:

The 67 kDa high-affinity laminin receptor was identified as an SINV receptor based on antibody blocking and enhanced binding in overexpression studies [34]. Whereas laminin receptors are involved in various cellular signaling and tissue repairs, dysregulations result in cancers and neurological disorders [34]. Even though biochemical and functional studies involving overexpression of laminin receptors in CHO and BHK cells increased SINV binding and susceptibility as well as antibodies against it inhibited infection, direct binding to SINV, evidence of receptor-mediated internalization, and information on direct interaction between the virus and ligand are lacking. A similar study carried out using a monoclonal antibody suggested an interaction between the VEEV E2 protein and the C-terminal domain of the laminin receptor, but this interaction needs further validation [35]. Therefore, while strongly implicated, the laminin receptor’s definitive role in alphavirus entry remains to be fully established.

Prohibitin-1 (PHB1):

Prohibitin-1 (PHB1), a protein involved in cell proliferation and mitochondrial integrity, was proposed as a receptor for CHIKV after it was identified as a top hit by mass spectrometry following a two-dimensional virus overlay protein binding assay (VOPB) assay as a binding partner of CHIKV in microglial cells [36]. The role of PHB1 was further confirmed by demonstrating a reduction in CHIKV infection in vitro when antibodies were used to block PHB1, or siRNA was used to knock down PHB1. Moreover, colocalization and co-immunoprecipitation studies of infected cells demonstrated the interaction of PHB-1 and CHIKV E2 proteins. Whereas these results pointed to PHB1’s role in viral entry, they were unable to validate the direct binding of CHIKV to recombinant purified PHB1, underlining the need to further evaluate PHB1’s role in viral binding and internalization.

CD147 complex:

CD147 complex, consisting of five subunit glycoproteins, was identified as a potential CHIKV entry receptor following an affinity purification-mass spectrometry (AP-MS) analysis of CHIKV VLP-infected 293T cells [37]. Subsequent CRISPR/Cas 9-based knockout screens revealed that knockouts of individual components of the CD147 complex had a moderate to no effect on CHIKV infectivity. However, simultaneous knockout of SLC1A5 alongside CD147 or CD98 significantly decreased viral infectivity [37]. These findings collectively indicated the role of the CD147 protein complex in the CHIKV entry process. However, the extent of reliance on specific components of this complex varies among different alphaviruses. For instance, the replication of the arthritogenic viruses ONNV, MAYV, and RRV appears to be primarily dependent on SLC1A5, whereas no role of the CD147 complex was reported for VEEV infection. Additional experiments demonstrating direct interaction between CHIKV and the proposed CD147 complex protein member are warranted for them to be established as genuine receptors.

Macrophage Receptor with Collagenous Structure (MARCO):

Macrophages play an essential role in limiting viremia and viral dissemination of alphaviruses. SR-A6 MARCO, a member of the class A scavenger receptor family, features a characteristic cysteine-rich extracellular domain that consists of a collagen-like domain and a scavenger-receptor cysteine-rich (SRCR) domain [38]. SR-A6 MARCO has been indicated as a CHIKV receptor for viral uptake in macrophages [39]. Additionally, the deletion of MARCO and mutagenic analysis of E2 confirmed MARCO-mediated viral uptake in macrophages and pinpointed lysine residues of E2 as critical for mediating viral clearance in CHIKV, ONNV, and RRV [40]. In a more recent study, the same group identified SRCR-mediated binding and internalization of CHIKV, ONNV, and RRV [40]. Using monoclonal antibodies against the SRCR domain and ectopic expression of SRCR, they demonstrated that the SRCR domain is responsible for viral internalization. In addition to the previously identified lysine residues of E2, other basic and acidic amino acids on E2 and E1 were mapped that played a significant role in viral clearance [40]. Moreover, using chimeric MARCO wherein the SRCR domain was replaced from different vertebrate hosts, the species-specific role of the SRCR domain in viral internalization and its biological competence as an amplification host was determined. A detailed mutagenic analysis of the binding interface, along with a demonstration of direct binding of the SRCR domain with the virion, will help establish MARCO as a genuine receptor.

B. Bona fide receptors

Natural resistance-associated macrophage protein (NRAMP):

NRAMP is a broadly expressed cell membrane protein involved in transporting divalent metal ions, including iron transport. In eukaryotes, NRAMP transports metal ions across the plasma and endosomal membranes. They are broadly classified into two groups: NRAMP1 is restrictively expressed on macrophages and intracellular compartments, whereas NRAMP2 expression is ubiquitous [41]. Using a genome-wide RNAi screen of the *Drosophila* cell line, dNRAMP was identified as a receptor for SINV binding and entry in insects and found indispensable for infection of adult flies [42]. Furthermore, the roles of mammalian homolog NRAMP2 in SINV binding and entry were assessed using an NRAMP2-deficient cell line, demonstrating the essential role NRAMP2 plays in SINV binding and entry in mammalian hosts. Based on the sequence conservation of the extracellular loops in NRAMP among mammalian and insect hosts, it was hypothesized that SINV binds to these loops. However, the role NRAMP2 plays in other alphaviruses, along with structural details when complexed with viruses, needs to be further characterized.

Apolipoprotein E receptor 2 (ApoER2)

ApoER2 is a member of the LDL receptor family involved in the endocytosis of lipoproteins, most prominently apolipoprotein E (ApoE), and other ligands. ApoER2 was identified as part of the alphavirus receptor family along with VLDLR (discussed in Section 3.2.2), when blocking the VLDL receptor blocked SFV infection but not that of other alphaviruses, suggesting the usage of other members of the LDL receptor family [43]. Ectopic expression of ApoER2 ligand binding domains (LBD) in K562 cells devoid of either VLDLR or ApoER2 supported the infection of SFV, EEEV, and SINV, which was demonstrated to be inhibited by the addition of a soluble decoy receptor. Additionally, the infectivity of SFV and EEEV increased significantly in mice ectopically expressing ApoEr2, establishing it as a genuine alphavirus receptor [43].

#### 3.2.2. Structurally Characterized Receptor Complexes with Alphaviruses

Matrix remodeling associated 8 (MXRA8):

Matrix remodeling associated 8 (MXRA8) is a type I transmembrane protein with two Ig-like extracellular domains arranged in a unique head-to-head configuration, stabilized by intramolecular disulfide bonds that also define their tertiary structure as revealed by the crystal structures of human and mouse MXRA8 [17]. As a conserved cell surface receptor across vertebrates and expressed on multiple cell types, MXRA8 was identified as an entry receptor for CHIKV and SFV complex viruses [17,44]. MXRA8 mediates viral entry by binding to the ‘cleft’/‘canyon’ region of envelope glycoproteins, a process involving its D1, D2, and hinge regions of MXRA8, with its extracellular stalk mediating different modes of interactions [45].

**Figure 4:**
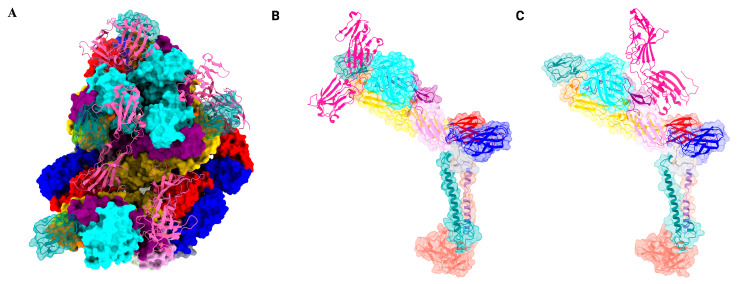
Mxra8 interaction with CHIKV E1-E2 glycoproteins. (**A**) *Left*, Top view of one asymmetric unit bound to four Mxra8 molecules (pink ribbon) occupying the cleft formed by the E1-E2 heterodimers. (**B**) *Middle*, Side view of a wrapped heterodimer wherein the MXRA8 makes contact with fusion loop (orange). (**C**) *Right*, Side view of MXRA8 making contact with heterodimer from adjacent spike (interspike). Two hundred and forty molecules of MXRA8 bind to each heterodimer in a stoichiometric ratio. Capsid protein is shown in light blue. The images were generated in ChimeraX Version 1.8 software using [46] and PDB ID: 6NK6.

Cryo-EM analysis of a CHIKV VLP in complex with MXRA8 revealed that each MXRA8 molecule occupies the ‘cleft’ region of the spike formed by the E2 A and B domain interface, establishing distinct binding site interactions with two adjacent E2-E1 protomers (Figure 4). Additionally, a 3:3 binding stoichiometry between MXRA8 and the E2-E1 trimeric spike complex was observed with three interaction interfaces described: ‘Wrapped,’ involving the E1 fusion loop (Figure 4B); ‘Intraspike,’ where adjacent E2 domains A and B are engaged (Figure 4C); and ‘Interspike,’ involving contacts between adjacent trimers [39,46]. Interestingly, the membrane distal domain D1 of MXRA8 interacts with the solvent-exposed E2 domain, whereas the D2 domain engages E1 proteins closer to the viral membrane.

Of note is the exclusive utilization of avian MXRA8 by the members of the WEE complex and not by the CHIKV and SFV complex members. WEEV and SINV engage avian MXRA8, however, the domain engagement of MXRA8 is inverted, interacting with different residues compared to the mammalian counterpart. The flipped orientation of MXRA8 notably resulted in the involvement of membrane distal D1, exclusively binding with E1, with E2 minimally involved [44]. Cryo-EM analysis of the complex of duck MXRA8 and WEEV VLP showed that D1 contacted MXRA8 at four sites of WEEV E1-DIII. Mutating or inserting amino acids in duck D1 at the predicted contact sites significantly reduced SINV infection in Δ*MXRA8* 3T3 cells complemented with duck MXRA8, validating the structural model. However, disruption of the predicted interspike contact in duck D1 had no impact on SINV infection, suggesting these interspike contacts, crucial for other alphaviruses, are less important for WEEV complex member infection mediated by duck MXRA8. It was further noted that mutations in duck D2 did not affect SINV infection [44].

Cattle MXRA8 has a 15-aa long insertion, forming a β-hairpin loop (the so-called “moo loop”) in its ectodomain. The moo loop sterically hinders binding by clashing with the E2-A domain residues implicated in MXRA8 engagement. This resulted in a failure of cattle MXRA8 to support viral infection in cell culture [47]. The infectivity in cell culture can be restored if the insertion in MXRA8 is deleted. Reciprocally, if the insertion was made in mouse MXRA8, it inhibited viral binding [47].

Low-density lipoprotein receptor class A domain-containing 3 (LDLRAD-3)

Low-density lipoprotein receptor class A domain-containing 3 (LDLRAD3) is a type I membrane protein belonging to the LDL scavenger receptor superfamily, which is predominantly expressed in neuronal tissues, skeletal muscles, and pancreas and is involved in regulating E3 ubiquitin ligase activity [48] and amyloid precursor protein (APP) processing [49]. As the name suggests, LDLRAD-3 has three extracellular domains: D1, D2, and D3, with D1 being the most distant from the membrane. The three domains consist of 30–40 amino acids, cysteine-rich repeats stabilized by disulfide bonds, and have calcium-binding properties attributed to the acidic amino acids, which contribute to the structural rigidity and functional properties of these domains. Whereas charged/polar residues dominate the surface, contributing to ligand binding/protein–protein interactions, the core of the domain is rich in hydrophobic residues [50].

A genome-wide CRISPR/Cas9-based screen identified LDLRAD3 as a receptor for the VEEV serocomplex. Genetic studies involving combinations of domain truncation mutants of LDLRAD-3 identified domain 1 of LDLRAD3 as necessary and sufficient for VEEV infection [51]. Furthermore, a direct binding of LDLRAD3(D1) with VEEV p62-E1 was confirmed. Additionally, the truncated version of LADRAD3(D1) supported VEEV infection in a neuronal cell line devoid of heparan sulfate [51]. Similarly, utilizing *Ldrad3*-deficient mice, Kafai et al. demonstrated the need for LDLRAD3 for efficient invasion of the central nervous system and disease progression [52].

**Figure 5:**
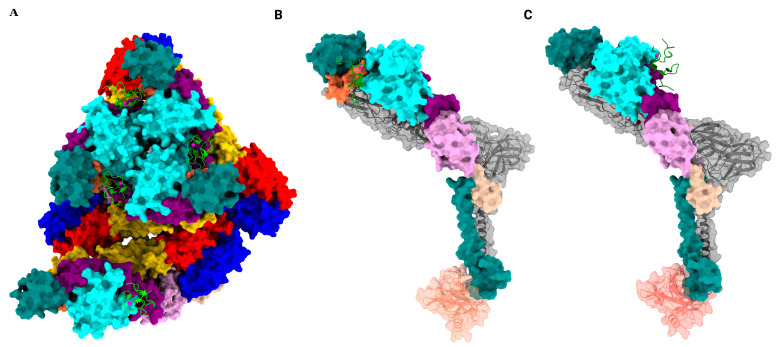
LDLRAD3 bound to VEEV. (**A**) *Left*, Top view of one asymmetric unit of VEEV bound to four molecules of LDLRAD3 (green ribbon). LDLRAD3 (D1) inserts itself into the cleft formed by two adjacent E1-E2 heterodimers and engages the fusion loop, E2-A, and B domains. (**B**) *Middle*, Side view of the wrapped heterodimer wherein the D1 of LDLRAD3 also makes contact with the fusion loop (orange). (**C**) *Right*, Side view of the interspike heterodimer showing contact with the β-ribbon connector and E2-A domain. The capsid protein is shown to be light blue. The images were generated in Chimerax version 1.8 using [53] and PDB ID: 7N1H.

Two high-resolution cryo-EM structures of VEEV-VLP complexed with LDLRAD3 were published concurrently (Figure 5). Three LDLRAD3(D1) molecules bound per trimeric spike, with each one of them wedged into a cleft formed between two adjacent E2–E1 heterodimers, resulting in 100% occupancy of the available sites. The binding resulted in a slight widening of the cleft and local conformational changes at the interface, the physiological significance of which remains to be investigated [53,54]. The interacting interface of LDLRAD3(D1) buried in the cleft was approximately 900–1000 Å^2^, almost half of what was observed for MXRA8 (~2100 Å^2^) [45,46] with interactions stabilized by hydrophobic patches, salt bridges, and hydrogen bonds [53,54]. The D1 domain engages E2 domains A and B and the E1 fusion loop at the wrapped heterodimer (Figure 5B). However, domain A and the β-linker play a pivotal role in stabilizing the interaction at the intraspike heterodimer (Figure 5C) [53,54]. In EEEV and WEEV, the residues in the binding interface of E2 are not fully conserved, thus leading to the inability of these encephalitic alphaviruses to engage LDLRAD3. However, further structural analysis of the i3 vs. q3 spikes bound to LDLRAD3(D1) did not show any significant difference in binding mode [54], with four distinct sites in the icosahedral asymmetric unit bound to the ligand reaching a full occupancy [53].

The hypothesis that the receptors like MXRA8 and LDLRAD3 might be involved in endosomal fusion, arising from their distinctive binding mode with viral glycoproteins –specifically, the engagement of the fusion loop and shielding them from solvent access –warrants further investigation.

Very low-density lipoprotein receptor (VLDLR)

A CRISPR/Cas9 screen of SFV RVP-infected 293T cells suggested VLDLR as a cellular receptor for SFV. Subsequent genetic and biochemical experiments, including knockout studies in VLDLR-deficient cells, ectopic expression in refractory cell lines, and receptor blocking with antibodies, confirmed VLDLR’s role as a bona fide entry receptor for SFV [43]. Ectopic expression of either VLDLR in K562 cells, which exclusively express LDLR, resulted in varying degrees of infection by SFV, EEEV, and SINV [43]. VLDLR comprises an N-terminal ligand-binding domain (LBD) consisting of eight cysteine-rich LDL-receptor class A (LA) repeats that coordinate Ca^2+^ ions, three EGF-like repeats, a beta-propeller domain, and an O-linked sugar domain [14]. Deletion of the VLDLR LBD resulted in a significant reduction in SFV VLP entry into 293T cells. Furthermore, direct binding assays demonstrated interactions between SFV, EEEV, and SINV VLPs and the VLDLR LBD, suggesting a potential interaction between viral envelope glycoproteins and the LDL-RA repeats [43].

**Figure 6:**
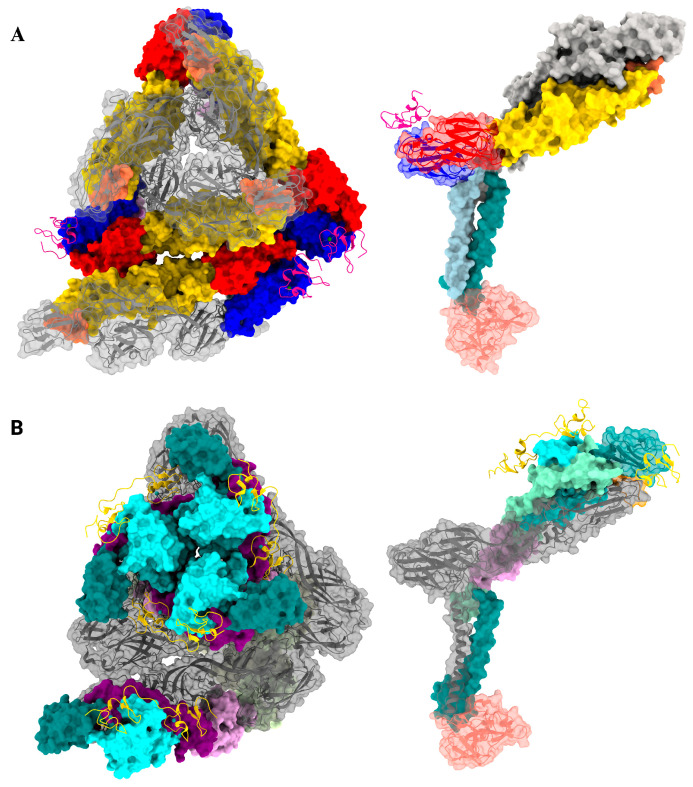
SFV and EEEV exhibit differential VLDLR binding mechanisms. (**A**) *Left*, Top view of one asymmetric unit of SFV bound to VLDLR molecules (pink ribbon) on the DIII (blue) of the E1 molecule. The VLDLR LA3 binds with higher affinity at a 2-fold axis compared to a 5-fold. *Right*, Side view of a heterodimer depicting the interacting interface between VLDLR and DIII of E1. (**B**) *Left*, top view of one asymmetric unit of EEEV bound to four molecules of VLDLR engaging the cleft. The LA1 and LA2 domains (yellow-ribbon) wrap around the cleft, engage the β-ribbon connector and E2-B domain. *Right*, Side view of a heterodimer showing the interacting interface of VLDLR and EEEV glycoproteins. The ligand molecules are shown as cartoons while the glycoproteins in surface view. The capsid protein is shown in blue. The images were generated in ChimeraX version 1.8 software using [14,15] and PDB ID: 8IHP (SFV) and 8UFA (EEEV).

A unique ligand and alphavirus binding mode was identified when the presence of additional densities at E1-DIII was observed in the cryo-EM analysis of SFV-VLP complexed with VLDLR LA1-8 (Figure 6A). These additional densities, which were prominent around the 2-fold and 5-fold axes, were subsequently confirmed to be LA3 [14]. Additionally, the binding efficiencies of the recombinant proteins generated from individual VLDLR-LA domains with E1-DIII were further analyzed. LA3 exhibited the highest binding affinity, suggesting that membrane-distal N-terminal LA repeats are both necessary and sufficient for SFV infection. The densities around 2-fold and 5-fold axes were further evaluated in the SFV-LA3 complex, demonstrating strong binding around the 2-fold axis compared to the 5-fold axis. Additionally, block-based reconstruction of the 2-fold and 5-fold axes revealed more ordered binding in the complex formed with LA3 alone vs. the VLDLR LA1-8. It was further concluded that LA3 could not simultaneously occupy all E1-DIII sites on either the 2-fold or the 5-fold axes because of the steric hindrances [14].

The binding interface between the LA3 and E1-DIII domains is stabilized by salt bridges, cation-pi interactions, and hydrophobic interactions. It was also demonstrated that the different VLDLR-LA repeats simultaneously bound to the E1-DIII protein at the 2-fold and 5-fold axes, suggesting a flexible binding mode that increases ligand affinity. However, the calculated buried surface area of 378 Å^2^ was roughly one-third of the LDLRAD-3-VEEV interaction and one-sixth of MXRA8, which could potentially explain the weak binding affinities observed in SINV and EEEV.

In contrast to the SFV-VLDLR (LA3) complex structure, the cryo-EM structure of the EEEV-VLDLR LA1-8 complex revealed a more commonly reported mode of binding involving the E1-E2 heterodimeric cleft [15] (Figure 6B). Additionally, the demonstration of engagement of the E1-DII, the β-ribbon connector, the back side of the E2-B domain, utilizing positively charged residues on E2 by the LA domains, suggested a similar binding mode of VLDLR [15,55]. By utilizing domain-swapped variants of VLDLR with LDLRAD-3, it was revealed that LA domains can be interchangeably used to bind to the virus, and no single domain is necessary or sufficient. Additionally, when individual domains of VLDLR were expressed to assess their effect in EEEV-PE6 infectivity, it revealed the redundant roles of domains LA1, 2, 3, 5, or 6, whereas LA4, 7, and 8 had no effect, further bolstering the notion of cooperativity among these domains in the binding process [15,55,56]. Additionally, when a complex of EEEV-VLDLR-LA (1–2) was analyzed structurally, it was noted that the LA1 engaged the cleft formed by the E1-E2 heterodimer, similar to that of VEEV/LDLRAD3 and CHIKV/MXRA8, contrary to LA2, which distinctively engaged the E2-A domain in an angular fashion. The contact interface contributed by LA1 encompasses the E1 fusion loop and the E2 HKR loop; however, it is relatively small compared to that of VEEV/LDLRAD3. Despite a smaller surface area of individual binding interfaces, SFV and EEEV exhibited a higher cumulative affinity for VLDLR due to the potential concurrent engagement of multiple LA domains compared to CHIKV/MXRA8 and VEEV/LDLRAD3-LA1 [51,52].

Even though EEEV and SFV employ a similar binding mechanism that involves the conserved sites in the LA domain, these distantly related alphaviruses bind the LDL-receptors via distinct sites on their envelope glycoproteins. Simultaneous binding of multiple LA domains involving both E2 and E1 glycoproteins is a characteristic feature of EEEV-LDLR complex, whereas engagement of multiple LA domains within a single site on E1 glycoprotein, possibly contributing towards the avidity of the interaction, characterizes SFV’s interaction with VLDLR. This distinct binding mechanism highlights the evolutionary flexibility in receptor binding, contributing to a broad host range of alphaviruses [15].

Protocadherin 10 (PCDH10)

WEEV, an historical cause of encephalitic outbreaks in humans and horses, has shown decreased virulence in recent decades, with the reasons for this “viral submergence” remaining unclear. Using CRISPR-Cas9, Li et al. identified protocadherin 10 (PCDH10) as a cellular receptor for WEEV [57]. PCDH10 is a member of the cadherin superfamily of cell adhesion molecules. These are type I membrane proteins whose extracellular domain consists of multiple cadherin repeats (EC) that interact with other cadherin molecules or ligands in a Ca^2+^-dependent manner [58]. PCDH10 comprises six EC repeats (EC1-EC6) and is stabilized by Ca^2+^ ions. Deleting EC1 from PCDH10, followed by ectopic expression of the construct, abolished WEEV infection in the target cells. However, overexpressing EC1 alone, but not EC2 or control, made cells susceptible to WEEV infection, confirming EC1 as the primary WEEV binding site on PCDH10. Additionally, Group A strains of WEEV engaged VLDLR and APoER2 for entry in K562 cells, implicating the redundant role of PCDH10 and LDLR family receptors in neuronal infection [59,60].

In a recent print [60], it was shown that the EC1 of human PCDH10 wedges into clefts between neighboring E2-E1 protomers at approximately a 45-degree angle relative to the spike’s threefold axis, without causing any major conformational changes in either the spike or the ligand. A significant surface area (~1500 Å^2^) was buried, illustrating extensive contacts of EC1 with both E2 and E1. This contact area was larger than that of alphavirus spikes with individual LDLR LA repeats but smaller than that of MXRA8. While PCDH10 EC1 utilizes one face to contact the E2-A β-ribbon residues, the opposite face interacts with the adjacent E2’-E1’ protomer through salt bridges and stacking interactions. Additionally, EC1 makes contact with the E1’ fusion loop. The contact residues, excluding the E1’ fusion loop, are not conserved across encephalitic alphaviruses, thus explaining the inability of EEEV and VEEV to bind PCDH10 [60]. Although cryo-EM analysis of WEEV Imperial 181 complexed with sparrow PCDH10-EC1 revealed a similar binding mode to that of WEEV bound to human PCDH10, the binding preference of Imperial 181 for sparrows was explained based on specific residue variations within the E1 fusion loop and associated pocket. These residue substitutions at key interaction sites between PCDH10 EC1 and the E1-E2 heterodimer, which explained the species-specific binding preference, offered insight into viral submergence [60].

Although WEEV outbreaks have been “submerged” in recent decades, their recent reemergence in the Americas is a reminder that this decline is not permanent. Since some ancestral WEEV strains effectively use VLDLR and ApoEr2 [60], current strains might adapt to efficiently engage these receptors, increasing their endemic/epidemic potential. Additionally, the fact that current strains cannot bind to PCDH10, a major neuronal receptor, complicates the understanding of the neurological effects of the disease and hampers progress in developing vaccines and therapies, as the development of soluble receptor decoys targeting receptors is confounded. Nevertheless, these new insights into the selective binding of neuronal receptors by different WEEV strains offer a glimpse into the emergence, submergence, and possible reemergence of these pathogens, helping us prepare for the next outbreak.

## 4. Host Cell Restriction Factors Implicated in Viral Egress

Alphaviruses budding and assembly processes are highly organized, resulting in the exclusion of most of the host cell membrane protein from budding sites [53,54]. However, recent studies have reported a few instances of involvement of host cell antiviral effector molecules and attachment factors that inhibit viral egress from the cells.

Bone marrow stromal antigen 2 (BST-2)/Tetherin

Bone marrow stromal antigen (BST-2) is a dimeric protein with an alpha-helical ectodomain anchored by a single transmembrane domain on the N-terminus and a GPI anchor on its C-terminus. BST-2/Tetherin is encoded by an interferon-stimulated gene and is localized to various cellular membranes [61]. Tetherin has been indicated in the inhibition of viral release by anchoring particles to the host cell membrane, potentially leading to endocytosis and degradation [62,63,64,65]. Ectopic expression of human tetherin in HEK293 cells showed a significant reduction in the release of SFV and CHIKV. Interestingly, while both long and short tetherin isoforms inhibited vesicular stomatitis virus (VSV) release, only the longer isoform inhibited SFV release [61]. Similarly, tetherin co-localized with CHIKV E1 protein when cells overexpressing BST-2 were infected with CHIKV-VLP. However, the nonstructural protein 1 (nsP1) of CHIKV counteracted the viral tethering by downregulating BST-2 expression [65].

Even though the egress inhibition of alphaviruses seems a unique area to explore, there have been very few studies providing structural insight into their interaction and the mechanistic aspects of inhibition. Immunoelectron microscopy of HIV-1-infected cells revealed that the BST-2 positions itself directly to nascent virions, resulting in the insertion of the GPI anchor into the viral envelope, and subsequent tethering of the viruses [66]. Moreover, deletion studies have also suggested the role of the ectodomain in viral anchoring. It would be interesting to understand how these receptors interact with alphaviruses structurally.

T-cell immunoglobulin and mucin domain-containing proteins (TIM-1)

In contrast to their role in aiding viral attachment (discussed in Section 3.1.1), a recent study revealed a role of TIM-1 in inhibiting CHIKV release from infected cells [67]. Utilizing TIM-1 knock-out cell lines, it was demonstrated that viral release from these cells is more efficient than the parental cell line [67]. Additionally, they were able to establish the inhibitory effect of TIM-1 on CHIKV release by saturating the PS-binding site of TIM-1 with liposomes and demonstrating the enhanced effect it has on viral release [67]. Similarly, TIM-1, -3, and -4 were implicated in the inhibition of HIV-1 release from the infected cells [68]. The unexpected finding of PS receptors’ ability to retain the viruses on the host cell surface can be well aligned with the observation of reduced in vivo pathogenicity of HS-adapted arthritogenic alphaviruses [69]. This could be due to the viral progeny being trapped at the budding site, resulting in less efficient viral release, or viruses being trapped in cells that are not conducive to productive viral infection.

Nonetheless, a thorough investigation into the roles of these antiviral effector molecules in viral egress remains crucial. Moreover, investigating the interaction of alphaviruses with these molecules during budding will offer a perspective on the interplay and balancing act between promoting viral entry vs. inhibiting egress. Additionally, it is intriguing to consider whether these interactions could further elucidate the asymmetry observed in these icosahedral viruses. It has been hypothesized that the asymmetry could arise from imperfect closure of the icosahedral shell, potentially due to unfavorable membrane curvature and steric clashes of glycoproteins during the final act of scission from host cells [70].

## 5. Antibodies Against Alphaviruses and Their Binding Mechanisms:

Isolation and characterization of antibodies elicited against alphavirus infections have been reported in the literature over the last several decades [71,72,73,74,75,76,77,78]. Many such antibodies have been isolated from survivors of alphavirus infections or mice infected with alphaviruses. These antibodies have helped define several immunogenic epitopes on alphaviruses. It is important to note that the antibodies broadly belong to two main classes: neutralizing and non-neutralizing. Although neutralizing antibodies are typically highly protective, several non-neutralizing antibodies have also shown promising therapeutic potential against alphavirus infections in vivo [79,80]

### 5.1. Structural Analyses of Alphaviruses in Complex with Fab Fragments of Neutralizing Antibodies

Structural characterization of epitope recognition of several neutralizing antibodies of both human and murine origins has been reported in the literature [81,82,83,84,85,86]. The structural and other biochemical and biophysical analyses have revealed that these neutralizing antibodies tend to target E2 domains A, B, and the part of the β-ribbon connector that links them. This is not surprising given the fact that E2 forms the outermost layer of the virion. The structural analyses of EEEV in complex with Fab fragments of murine neutralizing antibodies showed that the structural arrangement of the spikes has a direct consequence on the occupancy of the antibodies on E2 domains A or B, depending on the way they bind the epitope. For example, a radially binding (perpendicular to the viral surface) Fab on domain B does not have many steric clashes around it, while tangentially bound (parallel to the viral surface) ones would likely lead to clashes with neighboring Fabs. The situation is completely reversed for domain A binding antibodies. Since domain A is closer to the center of the trimeric spike, radial binding would invariably lead to clashes between the Fabs. On the contrary, domain B forms the tip of E2, and hence the situation is reversed for antibodies that target E2 domain B. These structural features suggest that a radially binding Fab of a neutralizing antibody on E2 domain B will have higher occupancy than a tangentially bound one, and the occupancy differences are reversed for domain A binding antibodies.

**Figure 7:**
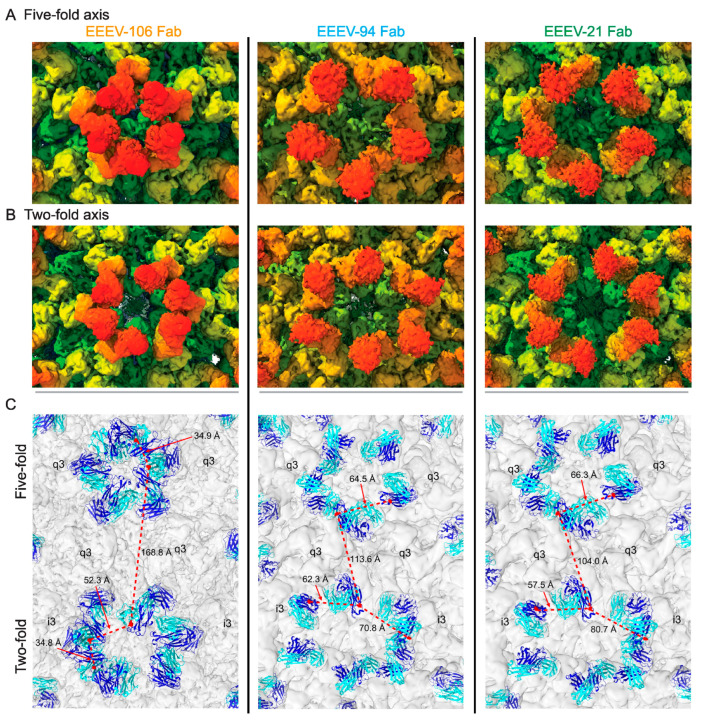
Human anti-EEEV arrangement at the icosahedral fivefold and twofold vertices on the viral surface. (**A**,**B**) *Left*, radially colored arrangement of EEEV-106, *Middle*, EEEV-94, or *Right*, EEEV-21 Fab fragments around the icosahedral fivefold (i5) or twofold (i2) axes of SINV/EEEV. (**C**) Distances (in Å) between the Ca atoms of the terminal heavy chain constant domain (CH1) cysteine residues (Cys216) of Fab neighboring Fabs [EEEV-106 (Left), EEEV-94 (Middle), or EEEV-21 (Right) at the i5 (Top) or i2 (Bottom) axes are shown for all three complexes by the dotted red lines and are indicated by the red arrows. The red spheres indicate the backbone atoms (N, Ca, C, and O) of Cys216. The heavy or light chains are colored blue or cyan, respectively. The q3 and i3 spikes are labeled in black, and the viral glycoprotein shell is faded for clarity. Figure adapted from [87].

Interestingly, alphavirus neutralizing antibodies that share overlapping epitopes can have significantly different neutralizing potencies. Structural analyses of Fab fragments of three anti-EEEV neutralizing antibodies, EEEV-106, EEEV-94, and EEEV-21, targeting overlapping E2 domain B epitopes, revealed that there appears to be a correlation between neutralization potency and the arrangement of the antibodies on the viral surface (Figure 7) [87]. Among the three antibodies studied, EEEV-106 is significantly more potent than the other two, with a greater than 20-fold difference in their respective IC_50_ values. The structural and biophysical analyses found that EEEV-106 formed intra-virion crosslinks and the other two formed inter-virion crosslinks, thereby aggregating the virus. The ability to form intra- or inter-virion crosslink on icosahedral viruses appears to be dependent on the distance between the C-α atoms of the last cysteine residues on the CH1 domains of the two Fab arms of the antibody being 50Å or less, since distances longer than 50Å would lead to structural instability of the disulfide bonds at the hinge region [88]. The correlation of neutralization potency and intra- or inter-virion crosslink formation by antibodies appears to hold also for neutralizing antibodies against flaviviruses [89]. This distance criterion that determines whether an antibody is likely to form intra- or inter-virion crosslinking is likely to be true for all icosahedral virus-antibody complexes.

It should be noted that, even though there are neutralizing antibodies that produce intra-virion crosslinks [87,90], there are no structures of individual monomeric Fab molecules crosslinking different protomers of either E2 or E1 glycoproteins. Interestingly, alphavirus E1 and flavivirus E protein share functional similarities, both lying roughly parallel to the viral membrane and being responsible for membrane fusion, there is plenty of structural information of neutralizing antibody Fab fragments crosslinking adjacent E proteins in flaviviruses [90,91]. An analogous situation in alphaviruses would be crosslinking of the E1 proteins at either icosahedral two-fold or five-fold by binding to both domains I and III. So far, such antibodies have not been structurally characterized. Most anti-E1 antibodies tend to be non-neutralizing. All the neutralizing antibodies against alphaviruses that have been identified and characterized target E2, which is not surprising since E2 forms the outermost shell of the virion, and is the primary immunogenic target for the humoral response [4]. Additionally, E2 shields much of the underlying E1. In principle, it seems likely that such antibodies should be present, but possibly at extremely low proportions, which makes identification very challenging.

Although numerous structural analyses of alphaviruses in complex with Fab fragments of neutralizing antibodies have been reported, there is only one reported structure of CHIKV in complex with an intact neutralizing murine IgG, CHIK-263 [92]. The structure shows that the two CHIK-263 IgG molecules crosslink spikes across the icosahedral two-fold. Owing to the relatively poor resolution of the reconstruction, the authors could not conclusively determine which two Fab arms constituted a single IgG molecule at the icosahedral two-fold. Additionally, there are poor densities around the icosahedral five-fold region that did not improve with localized reconstruction, which could indicate low occupancy. Here again, the authors referred to the distance criteria between the Fab arms as an important parameter that defined the binding mode of the antibody across the icosahedral two-fold. The distance between the epitopes at the icosahedral five-fold is different from that at the two-fold, the IgG crosslinks. Therefore, for the IgG to bind at the five-fold, the Fab arms have to move with respect to each other, which may be energetically expensive and could lead to weaker binding due to the possibility of a slightly different angle of approach to the epitope. This could be one of the reasons for the weaker IgG density observed at the icosahedral five-fold. Therefore, the binding and occupancy of an intact IgG versus the monomeric Fab fragments differ due to distance constraints between Fab arms governing intra-virion crosslinking and the epitopes borne out of the icosahedral arrangement of the alphavirus glycoproteins.

### 5.2. Pan-Arthritogenic Neutralizing Alphavirus Antibodies

Although several neutralizing antibodies have been characterized for several alphaviruses, antibodies that neutralize multiple arthritogenic alphaviruses, such as CHIKV, MAYV, RRV, and ONNV, have been isolated and characterized [93,94]. These antibodies, CHIK-265 and RRV-12, target two distinct but conserved and overlapping surface-exposed epitopes on E2 domain B. This region is an important target for immunogen design since it elicits pan-arthritogenic alphavirus antibodies. Unlike arthritogenic alphaviruses, neutralizing antibodies that target multiple encephalitic alphaviruses have not yet been isolated. Part of the reason could be because the sequence similarity of domains A and B of E2 proteins of encephalitic alphaviruses is lower in comparison to their arthritogenic counterparts.

### 5.3. Pan-Alphavirus Antibodies

Recently, human antibodies (DC2.112, DC2.315, EEEV-179) targeting pan-alpha epitopes on both arthritogenic and encephalitic alphaviruses on E1 glycoprotein have been isolated and characterized [79,95]. Although non-neutralizing, these antibodies offer protection in mice against infection from both arthritogenic and encephalitic alphaviruses. Mutational analyses, HDX-MS, and competition data showed that the epitope targeted by these antibodies lies on E1, adjacent to the fusion loop and at the base of the trimeric spike. These antibodies do not block viral entry or fusion, hence are non-neutralizing, but they block viral egress. The structural bases of epitope recognition by these antibodies have yet to be reported.

**Figure 8:**
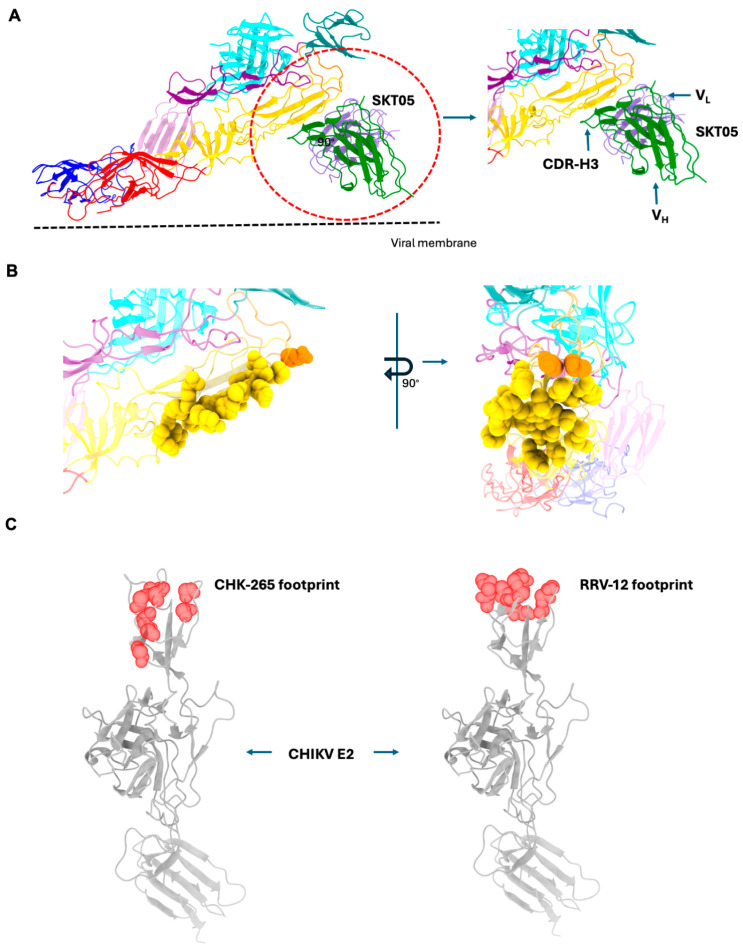
Pan-alpha epitope recognition by SKT05, CHK-265, and RRV-12. (**A**) *Left*, Cartoon representation of the VEEV E1/E2 heterodimer in complex with the Fab fragment of the SKT05 antibody (80, PDB: 8eeu). E1 and E2 are colored as in Figure 1 and Figure 2. Only the variable domains of SKT05 are shown, with VH and VL colored forest green and medium purple, respectively. The approximate position of the viral membrane is indicated by a dotted line. The antibody tangentially binds to the epitope. Right: Zoomed-in view of the interaction interface, with VH, VL, and CDR H3 all indicated. (**B**) *Left*, Residues contacted by the antibody are depicted as spheres and colored accordingly. The view is identical to that of the right panel in (**A**). The antibody is omitted for clarity. Right: Same as left but rotated around the indicated axis by 90°; these clusters of residues are adjacent to the fusion loop. (**C**) *left*, Cartoon representation of CHIKV E2 extracellular domain protein colored in gray with the residues recognized by neutralizing antibodies CHK-265 [92] and right, RRV-12 [93] shown as red spheres. E1 is not shown for clarity. These residues comprise an epitope conserved on several arthritogenic viruses.

Around the same time, murine pan-alpha antibodies that target E1 glycoprotein were reported along with structural characterization [80]. One of these antibodies, SKT05, targets the same region targeted by the human pan-alpha antibodies mentioned above. The authors present two structures of SKT05 in complex with WEEV and VEEV VLPs (Figure 8) [80]. The resolution of the two complexes, as reported in the paper, is 4.2 Å and 3.5 Å, respectively. Interestingly, no structure of SKT05 in complex with EEEV was reported. Given that the complex was formed by incubating the VLPs with the Fab fragment of SKT05 at room temperature, this epitope is accessible on the WEEV and VEEV VLP surfaces [80].

The structures revealed that SKT05 approaches its epitope in a tangential manner, i.e., the Fab fragment lies parallel to the VLP membrane. The Fab bound at the base of a q3 spike at the icosahedral two-fold region on the VLP surface. No binding was observed at the base of either the i3 spike or at the icosahedral five-fold region, likely due to steric clashes with neighboring spikes owing to the angle of approach of the Fab to the epitope. The residues engaged by SKT05 CDRs (Complementarity Determining Regions) are not fully conserved across the encephalitic and arthritogenic alphaviruses. Interestingly, the CDRs of SKT05 interact with the backbones of the sequence variable regions in the epitope, a novel but not unprecedented mode of recognition. Like DC2.112, DC2.315, and EEEV-179, SKT05 is also non-neutralizing and appears to offer protection in mouse models of alphavirus infection.

## 6. Implications for Vaccines and Therapeutic Development

The prevalence of alphaviruses across the vast geographical area, exacerbated by global travel and climate change-driven expanding vector habitats, poses a significant public health concern. Further, the selection potential of these viruses in adapting to new species of vectors, as exemplified by the E1 mutation (A226V) in CHIKV that enabled efficient replication in a new mosquito vector *Aedes albopictus* [96], with potential for massive outbreaks in geographically new regions, also underlines the need for the development of effective vaccines and therapeutics. With the recent approval of the VLP-based vaccine, VIMKUNYA (https://www.fda.gov/vaccines-blood-biologics/vimkunya (accessed on 30 May 2025)), along with the already available live attenuated IXCHIQ, against the highly pathogenic CHIKV, the relentless effort in understanding alphavirus biology is slowly coming to fruition. However, both these vaccines are age-restricted. Moreover, the ability of some of the encephalitic alphaviruses (EEEV, VEEV, and WEEV) to be aerosolized poses a different form of challenge, further exacerbated by the lack of approved vaccines for human use, even though several approaches have been adopted and tested to identify potential vaccine candidates [97].

The development of an effective vaccine against alphaviruses must consider multiple factors, including the biology of the viruses, their pathogenesis, tropism, and clinical outcomes, as well as the immunological responses that ensue. While the alphavirus genus harbors a unique group of structurally similar viruses, they present with different etiologies—encephalitic vs. arthritogenic. Even within these subgroups, different viruses, or more so different strains of the same viruses, can engage various types of receptors for cellular entry [8]. This promiscuity of receptor engagement can pose challenges to antibody-mediated protection for a subset of antibodies that target receptor engagement. However, antibodies that block fusion do not have this problem. The neutralizing antibodies against alphaviruses can block attachment, entry, or fusion, or all of them. In contrast, non-neutralizing antibodies tend to block viral egress. Additionally, safety and reactogenicity have been a persistent concern that has plagued some of the live attenuated alphavirus vaccines. For example, equids vaccinated with TC83 showed disease signs and symptoms, suggesting potential transmission into mosquitoes as well [98]. Since the attenuation depended on two point mutations, reversion to the wild-type could be potentially hazardous [99]. Similarly, the FDA and CDC recently recommended a pause on the use of the Ixchiq vaccine in individuals over sixty because of the reported severe adverse neurological and cardiac events, including two deaths, in vaccinated individuals within this age group (https://www.fda.gov/safety/medical-product-safety-information (accessed on 12 July 2025)). Despite these challenges, numerous alphavirus vaccine trials utilizing multiple vaccine platforms from live-attenuated to formalin-inactivated are at different phases of clinical trials [97]. Alphavirus VLPs, known to preserve the epitopes, which elicit robust immune responses, serve as yet another attractive platform for vaccine trials [80].

Characterizing the complete repertoire of attachment factors and receptors required for viral entry will help illuminate the key aspects of virus biology, tropism, and pathogenesis. Moreover, identifying the mode of binding and crucial residues involved in the interaction will guide the development of potential therapeutic interventions and enhance our understanding of the selection pressure these pathogens undergo to adapt. With the advent of the CRISPR/Cas9 system, several new bona fide alphavirus receptors have been characterized. High-resolution cryo-EM reconstructions of some of the clinically important alphaviruses complexed with their cognate receptors have unveiled the molecular basis of recognition of these viruses by their receptors. The structural information gleaned from these studies offers templates for therapeutic intervention. For instance, the MXRA8 binds to CHIKV, MAYV, and RRV, engaging the cleft formed by the envelope glycoproteins [45]. The authors then generated soluble versions of the receptor fused to the Fc-fragment, which acts as a decoy receptor and triggers an immune response. Interestingly, cryo-EM structures of LDLRAD3-VEEV [53] and VLDLR-EEEV [15] complexes showed that, similar to CHIKV-MXAR8, the receptors utilize the same cleft region in the envelope glycoprotein spike, opening the possibility of developing broad and potent inhibitors by targeting the cleft using structure-guided design strategies. Additionally, the discovery of a loop insertion in the ectodomain of cattle MXRA8 rendering many alphaviruses unable to bind and initiate infections [47] provides a platform to compare these receptors to other evolutionarily related receptors to understand how sequence variations modulate virus-receptor interaction and informs us towards the development of pan-alpha receptor inhibitors.

Structural studies of both human and murine antibodies targeting various sites on alphaviruses have defined the immunogenic sites of these viruses [100]. Additionally, the discovery of pan-alpha neutralizing and non-neutralizing antibodies has revealed these unique epitopes that are of significant therapeutic potential. These studies have opened two broad avenues for therapeutic intervention against alphavirus infections utilizing antibodies. The first is the use of patient-derived neutralizing antibodies directly after an infection or as a prophylactic in areas where alphavirus infections have broken out. In such situations, potently neutralizing antibodies highly specific against the causative alphavirus pathogen can be utilized to neutralize the virus. Additionally, pan-alpha neutralizing antibodies that target arthritogenic viruses offer a greater advantage given their ability to target several arthritogenic alphaviruses [93]. Pan-alpha antibodies that target both arthritogenic and encephalitic alphaviruses [79,80,101] are of great interest, given their ability to target both classes of viruses. However, these antibodies are non-neutralizing, even though they offer protection in in vivo studies against alphavirus infections. Ultimately, pan-alpha neutralizing antibodies that target both arthritogenic and encephalitic alphaviruses would be ideal for such situations, but none have been isolated so far.

The second avenue involves generating an immunogen that presents the selected epitopes in the correct conformation for use as vaccine candidates. Towards this end, utilizing the pan-alpha epitope is of great importance. Given the recent developments in the field of synthetic biology, it may be possible to generate scaffolds that stabilize these epitopes in the conformation that mimics their structure on the viral surface and therefore can be utilized as a vaccine candidate.

## 7. Conclusions and Future Directions

Alphavirus entry into cells involves a complex interplay of attachment factors, host cell receptors, and various host factors. Recent advances in alphavirus biology have identified several authentic viral receptors and characterized their roles in viral entry, addressing several key questions about viral tropism and viral submergence. These findings have significant implications for designing and developing countermeasures against these viruses. Furthermore, details of viral interaction with cellular receptors have opened up avenues for structure-guided therapeutic design targeting the receptor-recognizing regions on the viral surface. Even though there have been significant advances in understanding the molecular basis of alphavirus receptor and antibody recognition, several questions remain in this field, as listed below (Table 1), that merit further research.

## Figures and Tables

**Table 1:** Outstanding questions in Alphavirus receptor interaction and antibody response.

i.	How does the interplay of different attachment factors and receptors influence tissue tropism and viral entry?
ii.	Do other endocytic pathways play a role in alphavirus entry, and how do host factors influence the outcome?
iii.	Since SFV and EEEV bind VLDLR in two different modes, are there other alphavirus-receptor pairs that display different binding modes or engage multiple receptors?
iv.	Alphaviruses infect a wide range of hosts by engaging different receptors. Are there common receptors utilized by some alphaviruses in both mammalian and insect hosts that can be targeted?
v.	Since no pan-alpha neutralizing antibodies against both the encephalitic and arthritogenic viruses have yet been isolated, is it possible to utilize the structures of alphaviruses in complex with Fab fragments of pan-alpha non-neutralizing antibodies along with computational tools to develop neutralizing versions of these types of antibodies while the search continues for such antibodies from both human and murine sources?
